# Asprosin Protects H9C2 Cells From Ferroptosis Following Hypoxia/Reoxygenation by Promoting Mitophagy

**DOI:** 10.1155/cdr/8401037

**Published:** 2026-03-09

**Authors:** Xiangkun Wang, Xiaoyu Zhang, Huaiguang Tang, Xiaojuan Su, Shutong Ding, Xuelian Li, Bingong Li

**Affiliations:** ^1^ Department of Neurology, The Second Affiliated Hospital, Harbin Medical University, Harbin, Heilongjiang Province, China, hrbmu.edu.cn; ^2^ School of Clinical Medicine, Shandong Second Medical University, Weifang, Shandong, China; ^3^ Department of Cardiology, Union Hospital, Tongji Medical College, Huazhong University of Science and Technology, Wuhan, China, hust.edu.cn; ^4^ Department of Cardiology, Qingdao Hospital of Rehabilitation University, East Hospital of Qingdao Municipal Hospital, Qingdao, Shandong, China; ^5^ Department of Cardiology, Qingdao Municipal Hospital, Qingdao University, Qingdao, China, qdu.edu.cn

**Keywords:** asprosin, cardiomyocyte protection, ferroptosis, hypoxia/reoxygenation injury, mitophagy

## Abstract

**Background:**

Acute myocardial infarction is a leading cause of death globally. Percutaneous coronary intervention is the primary treatment to restore blood flow to the affected myocardium, but reperfusion can cause myocardial injury, affecting the prognosis of patients with acute myocardial infarction. Asprosin (ASP) is a newly discovered adipokine whose role in myocardial protection requires further research.

**Methods:**

The GSE240847 dataset was downloaded from the GEO database, and 511 ferroptosis‐related genes were collected from the FerrDb database. Gene coexpression network analysis (WGCNA) was performed to identify coexpression modules associated with Fibrillin 1 (FBN1), followed by enrichment analysis. H9C2 cells were subjected to hypoxia/reoxygenation (H/R) and pretreated with ASP at different concentrations. The effects of ASP were determined by measuring cellular reactive oxygen species (ROS), Cell Counting Kit‐8 (CCK‐8), and lactate dehydrogenase (LDH) levels and assessing the expression of ferroptosis‐related proteins, intracellular iron content, mitophagy‐related proteins, and mitochondrial membrane potential.

**Results:**

Enrichment analysis showed Gene Ontology (GO) terms linked to GTPase signaling, chromosome behavior, and cell stability. Kyoto Encyclopedia of Genes and Genomes (KEGG) analysis highlighted mitophagy and MAPK pathways in the FBN1 module. ASP cut ROS, boosted cell viability, and raised glutathione peroxidase 4 (GPX4)/solute carrier family 7 member 11 (SLC7A11) expression, upregulating glutathione and lowering iron particles dose dependently post H/R. It also increased PINK1 and stabilized mitochondria. A mitophagy inhibitor reduced these effects.

**Conclusions:**

This study confirms the protective effects of ASP on myocardial cells after H/R injury and demonstrates that ASP can inhibit ferroptosis and promote mitophagy in myocardial cells during ischemia–reperfusion injury. The potential mechanism may involve ASP promoting PINK1‐associated mitophagy in myocardial cells after H/R injury to inhibit ferroptosis.

## 1. Introduction

Acute myocardial infarction (AMI) is one of the leading causes of death globally, and percutaneous coronary intervention is the primary treatment method to restore blood flow to the affected myocardium. However, reperfusion can cause myocardial injury, which is a significant factor affecting the prognosis of AMI patients [1]. H/R triggers a series of adverse events, including excessive production of ROS, ferroptosis, calcium overload, inflammation, apoptosis, and mitochondrial dysfunction, leading to disruptions in cellular metabolism and energy supply. This ultimately results in cardiac cell death and myocardial injury [2, 3]. Currently, the factors influencing reperfusion injury are not fully understood, and identifying these factors could aid in discovering more strategies to protect heart function after reperfusion.

ASP, an adipokine identified in 2016, is encoded by the FBN1 gene and is expressed in various organs, including the heart [4, 5]. It enhances the therapeutic effect of mesenchymal stromal cells on ischemic heart disease [6], reduces ROS in cardiomyocytes, and protects the heart, significantly improving left ventricular function [7–9]. ASP also induces mitophagy in adipocyte mitochondria [10] and ASP absence activated AMPK/p38 signaling [11]. However, it remains unclear whether ASP is involved in ferroptosis and mitophagy in cardiomyocytes.

Ferroptosis is significant in myocardial ischemia–reperfusion injury (MIRI). It involves multiple pathways, including the antioxidant system, iron metabolism, and lipid metabolism [12–16]. Features of ferroptosis include lipid peroxidation product accumulation, intracellular iron accumulation, glutathione (GSH) depletion, and reduced GPX4 activity, leading to lipid membrane damage [17]. Inhibiting ferroptosis can help reduce MIRI, mainly through the SLC7A11/GPX4 pathway [18, 19]. In a recent study, ASP has been shown to effectively improve neuronal function following I/R injury [20]. However, whether ASP is involved in the regulation of I/R‐induced cardiomyocyte ferroptosis remains unclear.

Mitophagy refers to the process through which damaged or superfluous mitochondria are selectively eliminated via autophagy‐induced lysosomal degradation. Phosphatase and tensin homolog–induced kinase 1 (PINK1) is intimately involved in the regulation of mitophagy. Under normal conditions, PINK1 is cleaved by PARL and degraded. Under stress, PINK1 moves to the outer mitochondrial membrane, recruits Parkin, and targets damaged mitochondria for lysosomal degradation [21]. Although mitophagy is traditionally considered a protective mechanism, it is also recognized that while moderate mitophagy maintains mitochondrial health and preserves energy production, excessive mitophagy in the absence of mitochondrial biogenesis can be detrimental to cells [22]. Therefore, the specific role of mitophagy remains a subject of intense debate. However, it is still unclear whether ASP can protect cardiomyocytes after I/R by affecting mitophagy.

Here, we describe the effects of ASP pretreatment on H/R‐induced cell death. We found that ASP protected H9C2 cells after H/R, and cell viability increased with higher ASP concentrations. Moreover, ASP directly promoted mitophagy in cardiomyocytes after H/R and suppressed ferroptosis. We also investigated how ASP inhibits ferroptosis in cardiomyocytes by promoting mitophagy, finding that blocking this process reversed ASP′s protective effects and its inhibition of ferroptosis. Our study suggests that ASP plays a significant role in H/R‐induced myocardial cell injury by affecting mitophagy and ferroptosis.

## 2. Methods

### 2.1. Data Source

GSE240847 was downloaded from the Gene Expression Omnibus (GEO) database (http://www.ncbi.nlm.nih.gov/geo/). The expression data of 21 rats′ samples were obtained, containing left anterior descending (LAD) coronary artery ligation to establish myocardial ischemia models for 1, 6 (MI1h and MI6h), and 24 h (MI24h) along with Sham controls (Sham), and 1‐ and 6‐h ischemia followed by reperfusion (MI1h/R23h and MI6h/R18h) and each group was pretreatment with fenofibrate. We also collected 511 ferroptosis genes from the FerrDb database (http://zhounan.org/ferrdb/legacy/index.html), including 264 driver genes, 238 suppressor genes, and 9 marker genes (which is larger than the gene count (484), because of 27 multiannotated genes). NCBI‐GEO and FerrDb belong to international public databases, which are used to help researchers query and download genetic data.

### 2.2. Function and Pathway Enrichment Analyses and WGCNA

We utilized the “Sangerbox” online tool (http://www.sangerbox.com) to perform WGCNA analysis and identified coexpression modules related to FBN1. Following that, we employed the “Sangerbox” online tool for enrichment analysis of the said co‐expression module. Select GO function analysis and KEGG pathway enrichment analysis, with *p* value < 0.05 and FDR < 0.05 statistically significant and select the Top 10. The analysis items include biological process (BP), molecular function (MF), cell component (CC), and signal pathway.

### 2.3. Cell Culture

The H9C2 cell line, a myoblast cell line derived from embryonic rat heart tissue, was obtained from the Cell Bank of the Chinese Academy of Sciences, Shanghai, China. According to the recommendations of Shanghai Cell Bank, the cells were seeded at a density of 2 × 10^5^ cells/well in 6‐well plates and cultured in Dulbecco’s modified Eagle medium (DMEM) supplemented with 10% fetal bovine serum (FBS) under a humidified atmosphere of 95% air and 5% CO_2_ at 37°C.

### 2.4. H/R Protocol

H9C2 cells were cultured in DMEM with 10% FBS at 37°C for 24 h under standard conditions. After washing with phosphate‐buffered saline (PBS) twice, the medium was replaced with glucose‐free DMEM pre‐equilibrated under hypoxic conditions (1% O_2_, 5% CO_2_, and 94% N_2_) for 2 h to remove dissolved oxygen. HR was induced by placing the cells in a hypoxia chamber with 1% O_2_, 5% CO_2_, and 94% N_2_ for 6 h. Subsequently, the medium was changed to high glucose DMEM with 10% FBS and incubated under reoxygenation conditions (95% air and 5% CO_2_) at 37°C for 12 h. ASP was preadministered at three concentrations (50, 100, or 150 nM) for 12 h. In Ferrostatin‐1 (Fer‐1, 5 *μ*M) group and Mitochondrial Division Inhibitor 1 (Mdivi‐1, 50 *μ*M) group, Fer‐1 and Mdivi‐1 were concurrently treated with ASP [23, 24].

### 2.5. Measurement of Intracellular ROS

Intracellular ROS levels were assessed using 2 ^′^,7 ^′^‐dichlorodihydrofluorescein diacetate (DCFH‐DA) and dihydroethidium, purchased from Beyotime, Shanghai, China. Cells were incubated with 10 *μ*M DCFH‐DA or 5 *μ*M dihydroethidium in FBS‐free DMEM for 20 min at 37°C, followed by two washes with FBS‐free medium. Fluorescence was measured using a fluorescence microscope at an excitation wavelength of 488 nm.

### 2.6. Cell Viability Assay

Cell viability was evaluated using the CCK‐8 assay (Sigma‐Aldrich; Merck KGaA) and LDH assay (Promega Corp.). H9C2 cells were plated in 96‐well plates at a density of 5 × 10^5^ cells per well. CCK‐8 solution (10 *μ*L) was added to each well and incubated for 4 h before measuring the optical density (OD) at 450 nm. For LDH detection, LDH reagent was added to the collected culture medium and incubated for 30 min at room temperature, protected from light. The OD value was quantified at 490 nm using a spectrophotometer. Cell survival rates were calculated by comparing the OD values of the experimental groups to the control group in the CCK‐8 assays. Relative cell death rates were determined by comparing the OD values of the experimental groups to the control group in the LDH assays.

### 2.7. Assessment of Mitochondrial Membrane Potential Dynamics

Mitochondrial membrane potential was assessed using JC‐1 fluorescence (C2005, Beyotime). Cells were incubated with JC‐1 staining solution at 37°C for 30 min, followed by two rinses with JC‐1 staining buffer to remove excess stain. Fluorescence was observed and quantified using a fluorescence microscope at excitation/emission wavelengths of 490/530 nm (green) and 525/590 nm (red). The relative fluorescence intensities of JC‐1 monomers and aggregates indicate the mitochondrial membrane potential.

### 2.8. Measurement of GSH

GSH levels in H9C2 cells were measured using a GSH Detection Assay kit (Catalog Number ab138881; Abcam) according to the manufacturer′s instructions. Cells were seeded into 96‐well white‐walled multiwall luminometer plates at a density of 5 × 10^4^ cells/well.

### 2.9. Western Blotting

H9C2 cardiomyocytes were homogenized in precooled RIPA lysis buffer following various treatments. The supernatant was collected and mixed with protein buffer. Equal amounts of total protein (10 *μ*g per well) were separated by SDS‐PAGE and transferred to PVDF membranes. Membranes were blocked in 5% skimmed milk for 1 hour, then incubated with primary antibodies (PINK1, 1:1000; GPX4, 1:1000; SLC7A11, 1:1000; Parkin, 1:1000; and *β*‐actin, 1:1000) overnight at 4°C. After incubation with HRP‐conjugated secondary antibody (1:10,000, Proteintech) for 1 h at room temperature, the membranes were analyzed using a biological image analysis system (Bio‐Rad, United States). *β*‐Actin levels were used as a loading control to determine the relative expression levels of the target proteins.

### 2.10. Intracellular Fe^2+^ detection

Log‐phase H9C2 cells were seeded into 24‐well plates. After the indicated treatments, the medium was discarded and FerroOrange working solution was added. Following a 30‐min incubation, cells were observed and photographed under a fluorescence microscope, and the images were analyzed with ImageJ.

### 2.11. Statistics

Data are presented as the mean ± standard error of the mean (SEM). Sample numbers, representing biological replicates, are specified in the individual figure legends. All data were analyzed in a blinded manner. Statistical analyses were performed using GraphPad Prism software (Version 7.01). For comparisons between two groups, a Student′s *t*‐test was used. For comparisons among multiple groups, one‐way or two‐way analysis of variance (ANOVA) was employed, with Bonferroni′s or Dunnett′s multiple comparisons test for normally distributed data or the Mann–Whitney test for nonnormally distributed data. A *p* value of less than 0.05 was considered statistically significant.

## 3. Results

### 3.1. WGCNA of GSE240847

**Figure 1:**
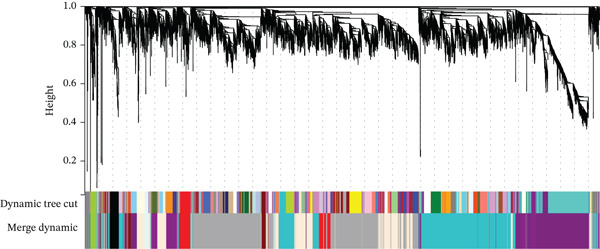
Clustering dendrogram of genes; various colors represent different modules.

To obtain a gene set with expression changes similar to those of the gene FBN1, which encodes ASP, during the ischemia–reperfusion process, we performed WGCNA analysis on the dataset GSE240847. First, we utilized gene expression profiles to calculate the median absolute deviation (MAD) for each gene. We excluded the Top 30% of genes with the smallest MAD and used the goodSamplesGenes method from the WGCNA R package to remove outlier genes and samples. We then constructed a scale‐free coexpression network with WGCNA. To classify genes with similar expression profiles into gene modules, average linkage hierarchical clustering was conducted according to the TOM‐based dissimilarity measure with a minimum size (gene group) of 30 for the genes′ dendrogram. The sensitivity was set to 3. To further analyze the module, we calculated the dissimilarity of module eigengenes, chose a cut line for module dendrogram, and merged some module. Additionally, we merged modules with a distance of less than 0.25, ultimately obtaining 11 coexpression modules (Figure 1). It is noteworthy that the gray module is considered a collection of genes that cannot be assigned to any module.

### 3.2. Function and Pathway Enrichment Analyses

We identified an FBN1‐associated coexpression module through WGCNA, which included 2161 genes, to verify the correlation of this module with mitophagy and ferroptosis; enrichment analysis was performed on it. For the GO analysis, the changes in BP included significant enrichment of several GTPase‐mediated cell signaling processes, such as regulation of GTPase activity, small GTPase–mediated signal transduction, and positive regulation of GTPase activity (Figure 2a). The changes in CC included significant enrichment related to chromosomal behavior and structural changes, such as chromosomal region, chromosome, centromeric region, and condensed chromosome (Figure 2b). The changes in MF included significant enrichment of functions related to cellular stability and energy provision, like DNA helicase activity, microtubule binding, and DNA‐dependent ATPase activity (Figure 2c). For the KEGG analysis, we found that the coexpression module where FBN1 resides was enriched in mitochondrial autophagy and MAPK signaling pathways, and the MAPK signaling pathway is related to both ferroptosis and mitochondrial autophagy. We also intersected the coexpression module where FBN1 is located with the ferroptosis‐related gene set, obtaining a total of 67 intersecting genes (including 40 driver genes, 30 suppressor genes, and two marker genes, with five overlapping genes).

Figure 2Enrichment function analysis of FBN1‐associated coexpression module. (a–c) GO enrichment in the FBN1‐associated coexpression module. (d) KEGG enrichment in the FBN1‐associated coexpression module. (e) Related genes of FBN1‐associated coexpression module and mitophagy pathway. (f) Venn diagrams of the FBN1‐associated coexpression module and ferroptosis‐related genes.

**Figure:**
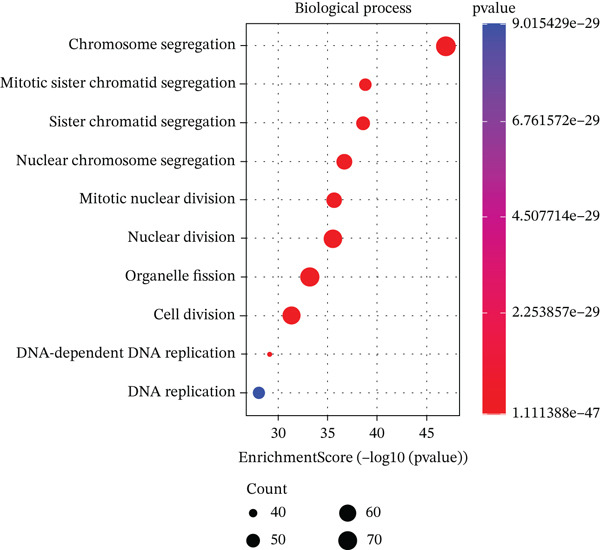
(a)

**Figure:**
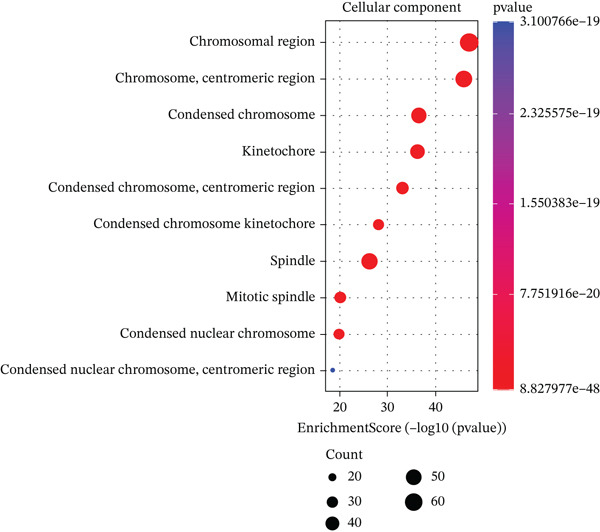
(b)

**Figure:**
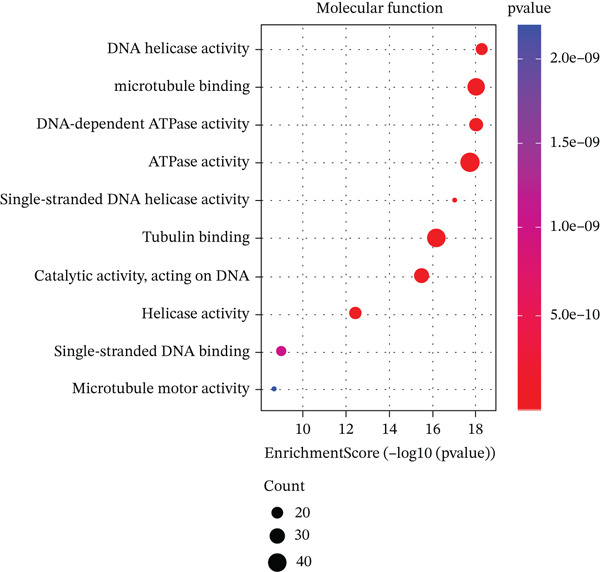
(c)

**Figure:**
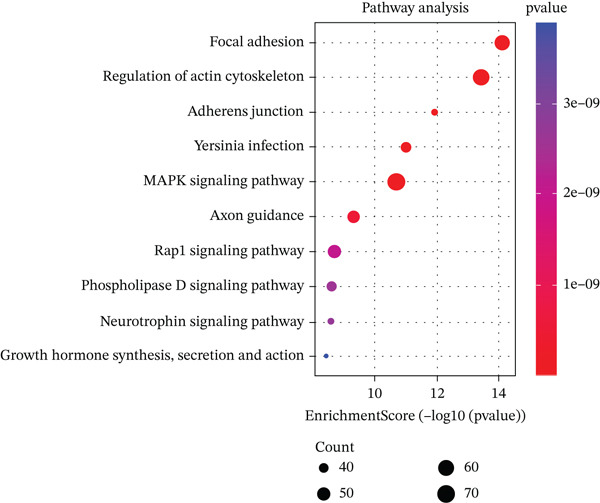
(d)

**Figure:**
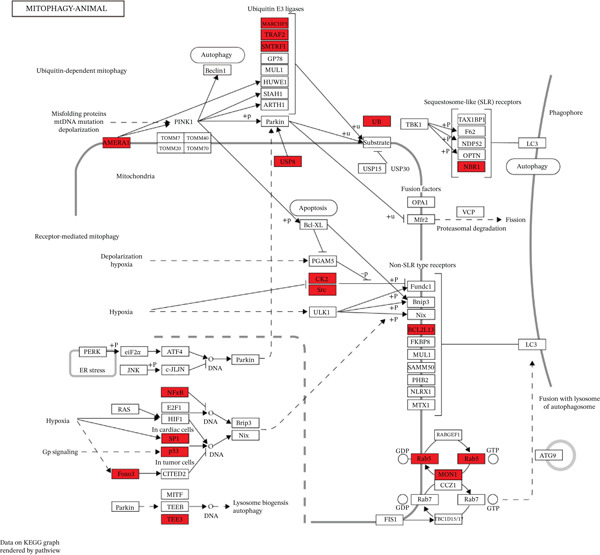
(e)

**Figure:**
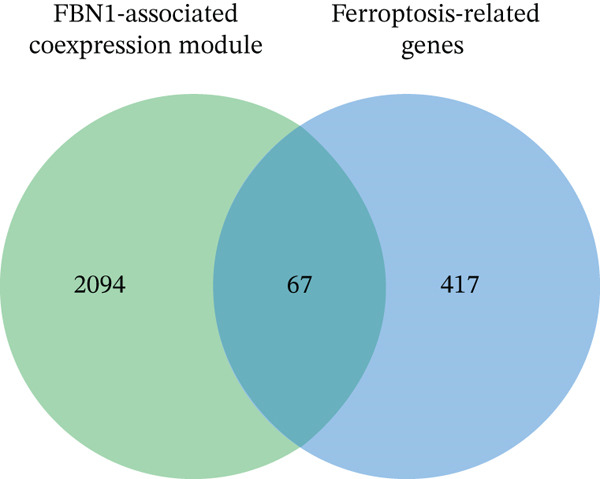
(f)

### 3.3. The Protective Effect of ASP on H/R‐Induced H9C2 Cell Injury

After pretreating H9C2 cells with a gradient of ASP (50, 100, or 150 nM) following H/R, ASP partially inhibited the elevation of ROS induced by H/R in H9C2 cells in a dose‐dependent manner (Figure 3a,b). Furthermore, we employed the LDH and CCK‐8 assays to determine cell viability. We observed that ASP exerted a protective effect on the decline in H9C2 cell viability induced by H/R in a dose‐dependent manner (Figure 3c,d).

Figure 3Protective effects of ASP on H/R‐injured H9C2 cells. (a, b) After pretreating H/R‐injured H9C2 cells with a concentration gradient of ASP (50, 100, and 150 nM), the intracellular ROS concentration was determined using the corresponding quantitative detection kit (magnification 200x, scale bar 250 *μ*m). (c) After pretreating injured H9C2 cells with a concentration gradient of ASP (50, 100, and 150 nM), cell viability was assessed using the CCK‐8 kit. Cell viability was calculated by dividing the OD value of the experimental group by the OD value of the control group. (d) After pretreating injured H9C2 cells with a concentration gradient of ASP (50, 100, and 150 nM), LDH‐mediated cell death was determined. Data (*n* = 3) are presented as mean ± standard deviation, and significance is denoted as *p* < 0.05,  ^∗^ *p* < 0.05,  ^∗∗^ *p* < 0.01, and  ^∗∗∗^ *p* < 0.001.

**Figure:**
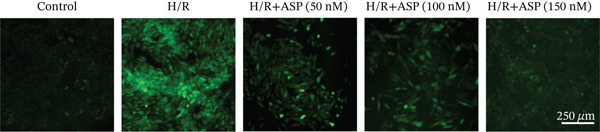
(a)

**Figure:**
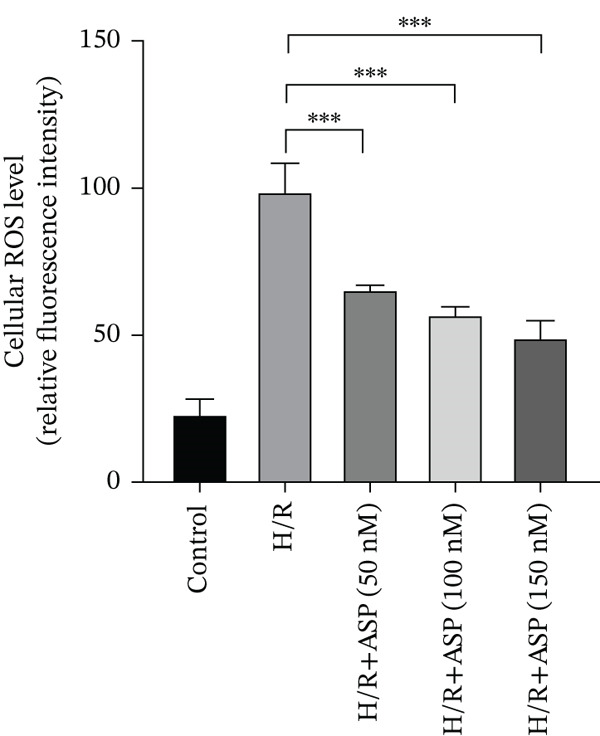
(b)

**Figure:**
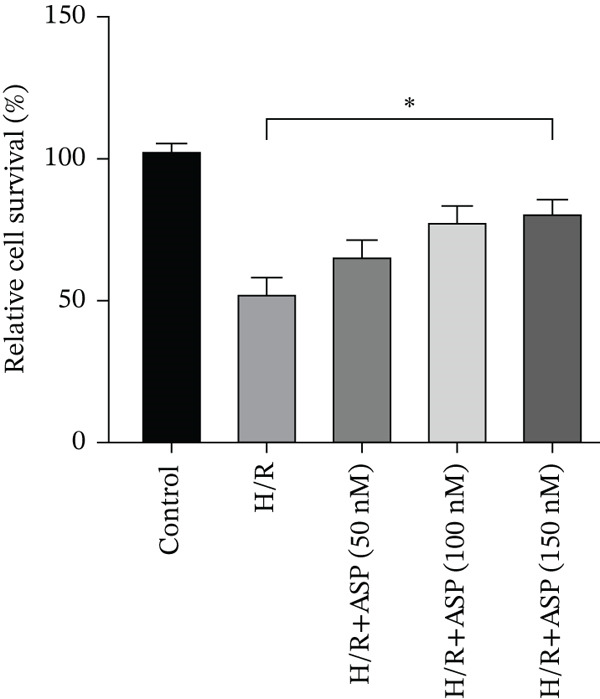
(c)

**Figure:**
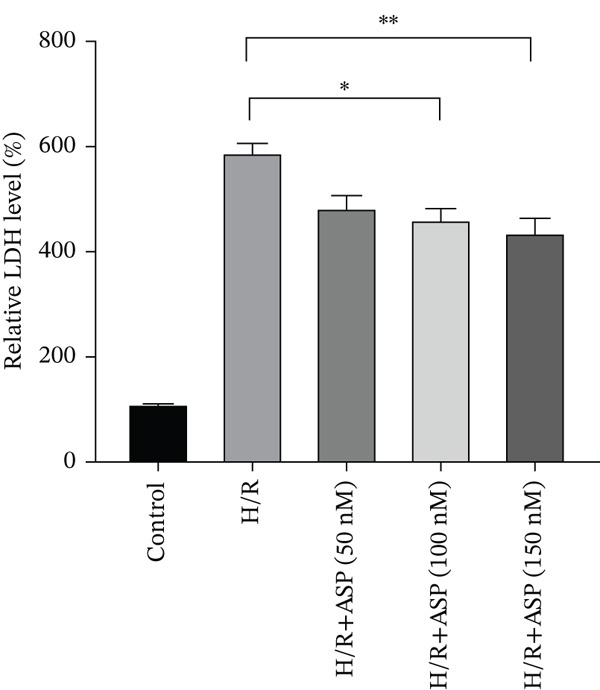
(d)

### 3.4. ASP Protects H9C2 Cells From Ferroptosis Induced by H/R

To further elucidate the protective role of ASP on H9C2 cells post‐H/R, we investigated the relationship between ASP and ferroptosis. We observed that ASP (50, 100, or 150 nM) partially alleviated the H/R‐induced decrease in the expression of GPX4 protein (Figure 4a,b) and SLC7A11 protein (Figure 4a,c) in a dose‐dependent manner and also inhibited the decline of GSH (Figure 4d). To further validate the protective effect of ASP on H/R‐induced H9C2 cells, we assessed changes in intracellular Fe^2+^ content. We found that ASP (50, 100, or 150 nM) partially reduced the H/R‐induced increase in intracellular Fe^2+^ content in a dose‐dependent manner (Figure 4e,f). To further confirm the ferroptosis inhibitory effect of ASP, we pretreated H9C2 cells with 100 nM ASP and included the ferroptosis inhibitor Fer‐1 (5 *μ*M) as a positive control group. We measured the ferroptosis‐related proteins GPX4 (Figure 4g,h) and SLC7A11 (Figure 4g,i), along with GSH levels (Figure 4j) and intracellular Fe^2+^ content (Figure 4k,l). These results suggest that ASP may inhibit ferroptosis in H9C2 cells induced by H/R.

Figure 4ASP is implicated in ferroptosis induced by H/R in H9C2 cells. (a, b) The expression levels of GPX4 were measured by western blotting. (a, c) The expression levels of SLC7A11 were measured by western blotting. (d) The impact of ASP at a concentration gradient (50, 100, and 150 nM) on the intracellular GSH content of H9C2 cells post‐H/R injury was assessed. (e, f) Intracellular Fe^2+^ content was determined using corresponding quantitative assay kits to observe the effect of ASP at a concentration gradient on Fe^2+^ in H9C2 cells following H/R injury (magnification 200x, scale bar 250 *μ*m). (g, h) GPX4 expression levels were determined by western blotting. (g, i) SLC7A11 expression levels were assessed by western blotting. (j) The effect of ASP on intracellular GSH content in H9C2 cells after H/R injury was evaluated under positive‐control conditions. (K, L) The effect of ASP on Fe^2+^ levels in H9C2 cells after H/R injury was assessed under positive‐control conditions (magnification 200x, scale bar 250 *μ*m). Data (*n* = 3) are expressed as mean ± SD, with significance indicated at *p* < 0.05,  ^∗^ *p* < 0.05,  ^∗∗^ *p* < 0.01, and  ^∗∗∗^ *p* < 0.001.

**Figure:**
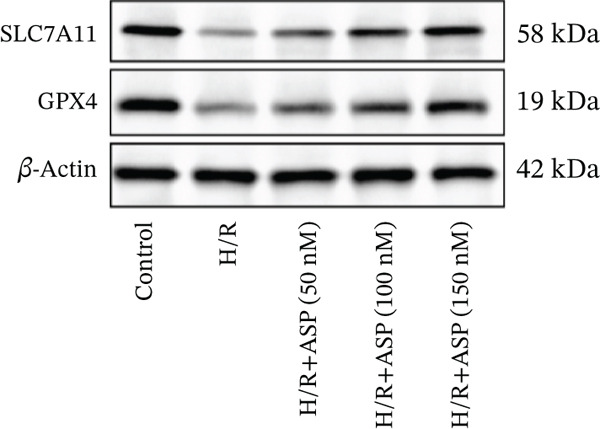
(a)

**Figure:**
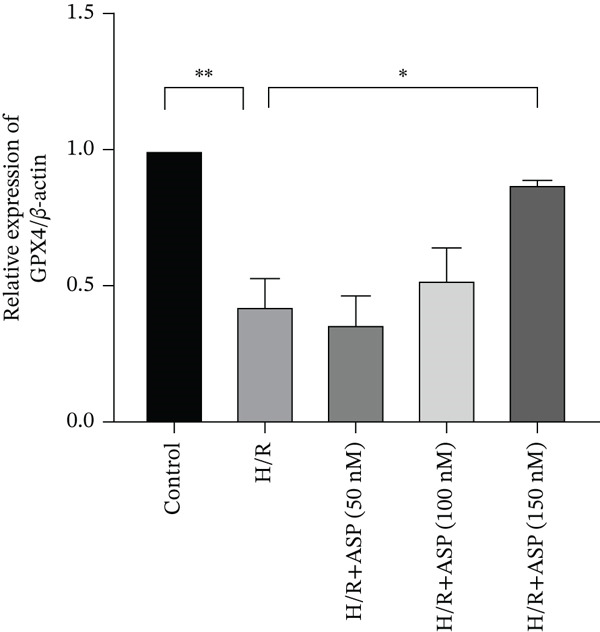
(b)

**Figure:**
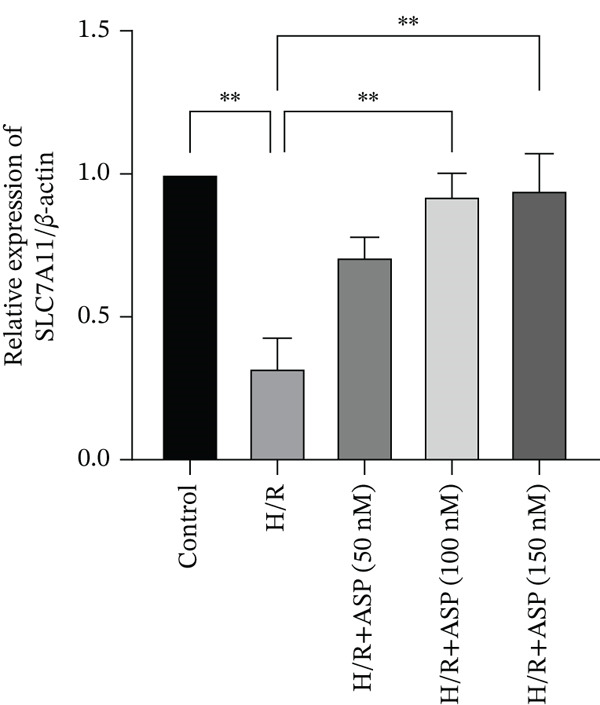
(c)

**Figure:**
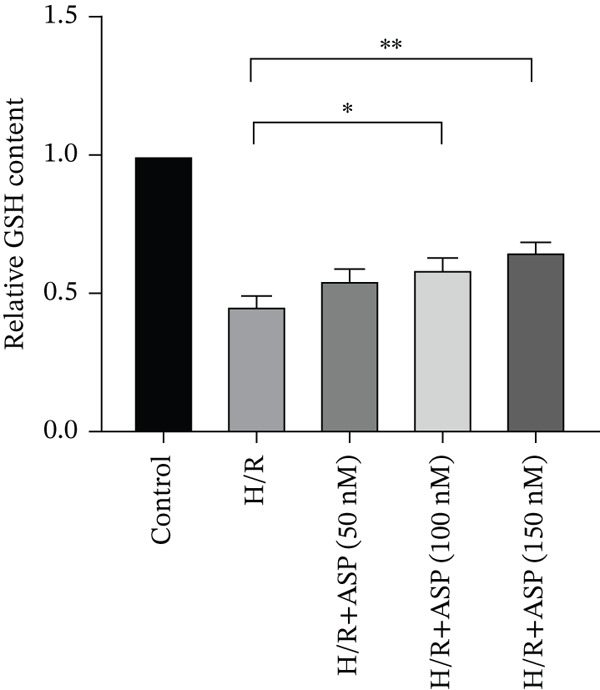
(d)

**Figure:**
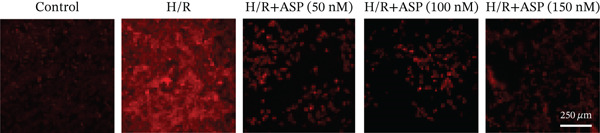
(e)

**Figure:**
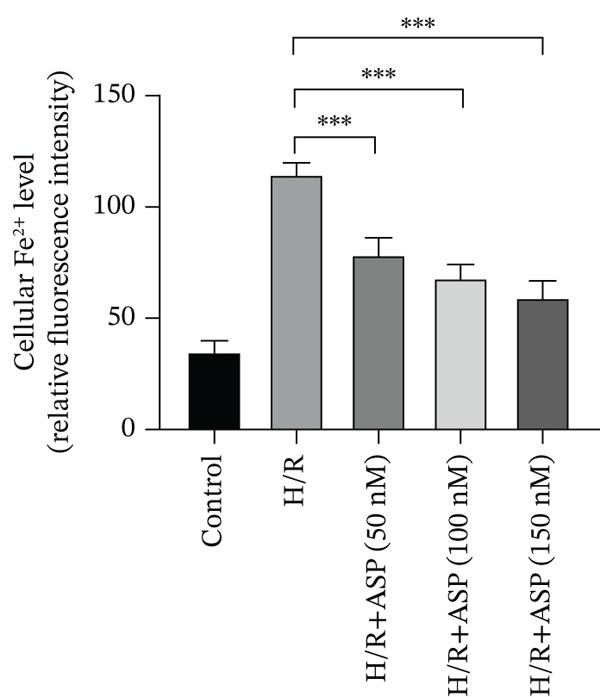
(f)

**Figure:**
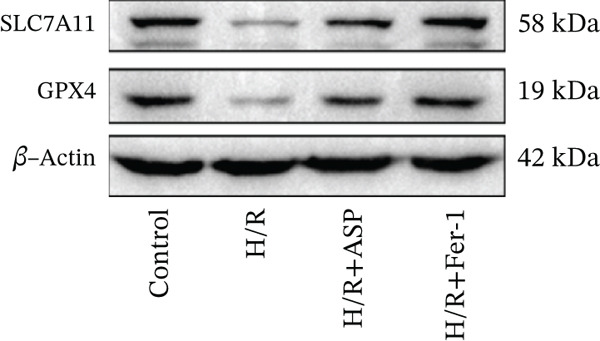
(g)

**Figure:**
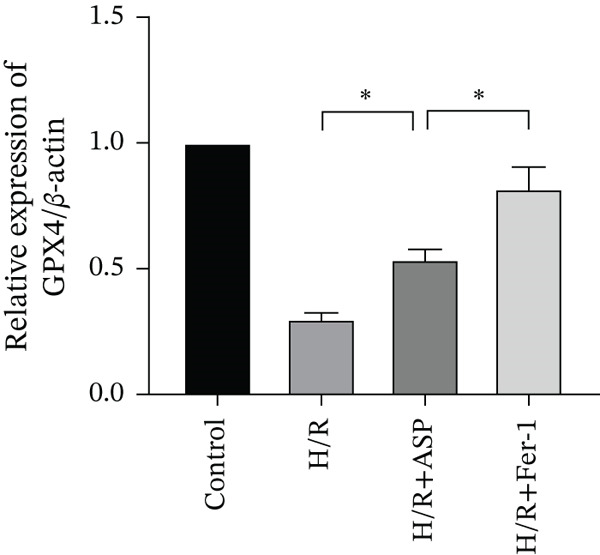
(h)

**Figure:**
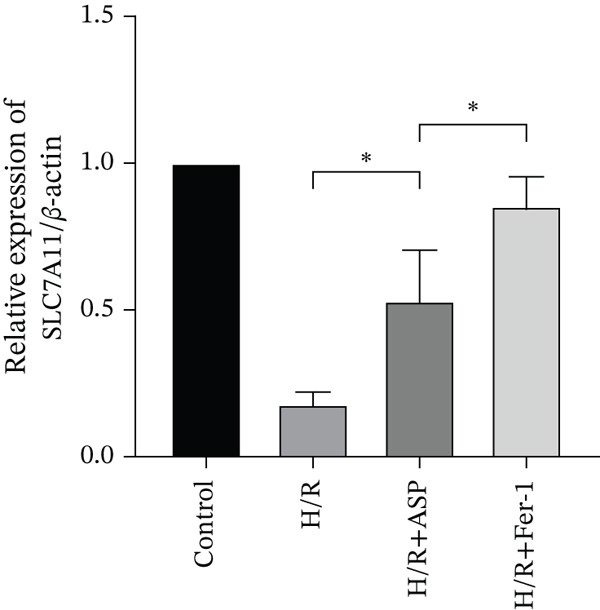
(i)

**Figure:**
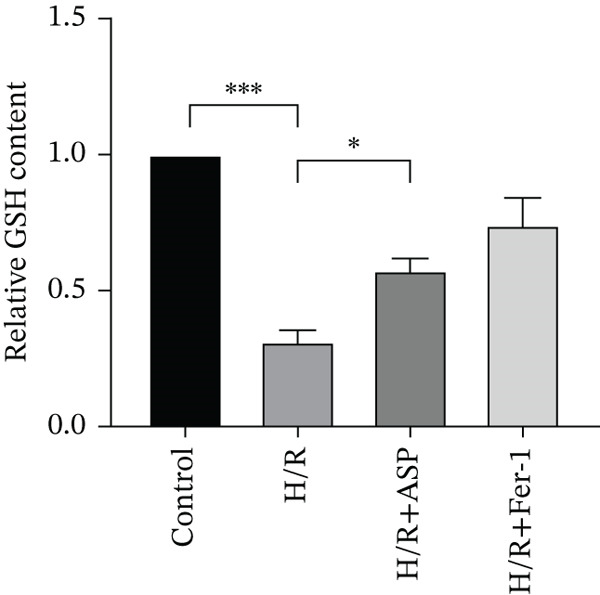
(j)

**Figure:**
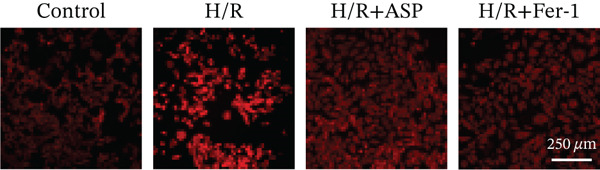
(k)

**Figure:**
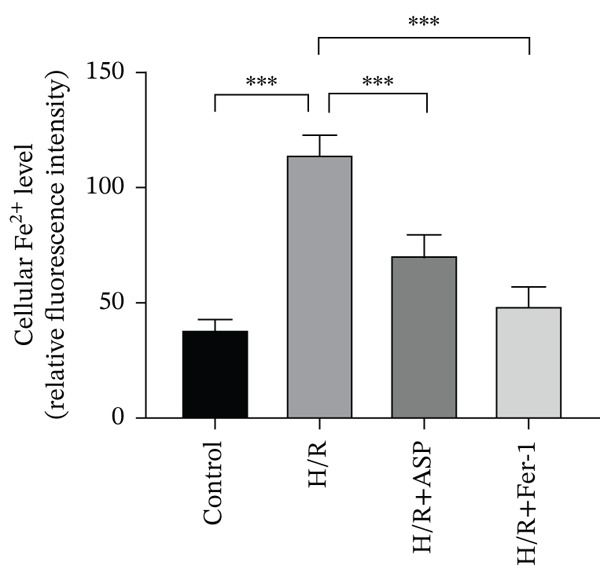
(l)

### 3.5. ASP Promotes Mitophagy in H9C2 Cells Following H/R Injury

Previous studies have indicated that ASP can promote mitophagy in cells. However, research on its role in cardiomyocytes is insufficient. We observed that ASP (50, 100, or 150 nM) dose dependently inhibited the expression of P62 protein and enhanced the expression of PINK1 and Parkin protein in H9C2 cells following H/R injury (Figures 5a, 5b, 5c, and 5d). Then, we determined the changes in mitochondrial membrane potential by JC‐1 staining. The results demonstrated that with the increase in the concentration of ASP pretreatment, the increased green fluorescence in H9C2 cells after H/R injury was reduced as the concentration of ASP increased (Figure 5e,f).

Figure 5ASP is involved in H/R‐induced mitophagy in H9C2 cells. (a, b) The expression levels of P62 were measured by western blotting. (A, C) The expression levels of Parkin were measured by western blotting. (a, d) The expression levels of PINK1 were measured by western blotting. (e) Fluorescence of H9C2 cell mitochondria JC‐1 staining (magnification 200x, scale bar 250 *μ*m). (f) Ratio of JC‐1 staining green/red fluorescence. Data (*n* = 3) are expressed as mean ± SD, with significance indicated at *p* < 0.05,  ^∗^ *p* < 0.05,  ^∗∗^ *p* < 0.01, and  ^∗∗∗^ *p* < 0.001.

**Figure:**
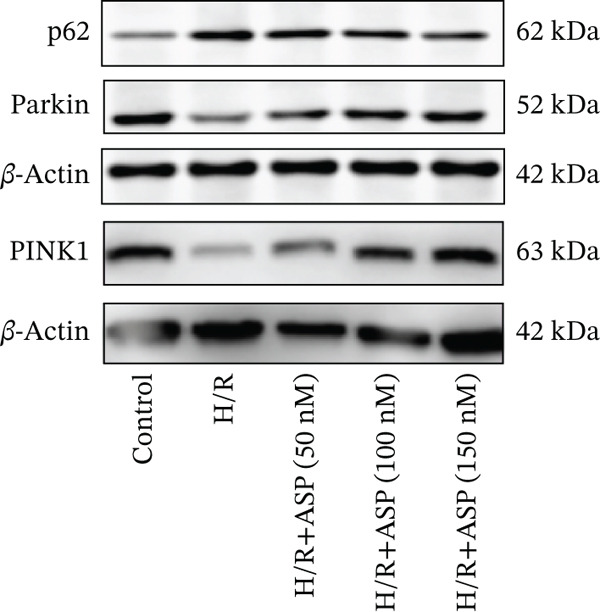
(a)

**Figure:**
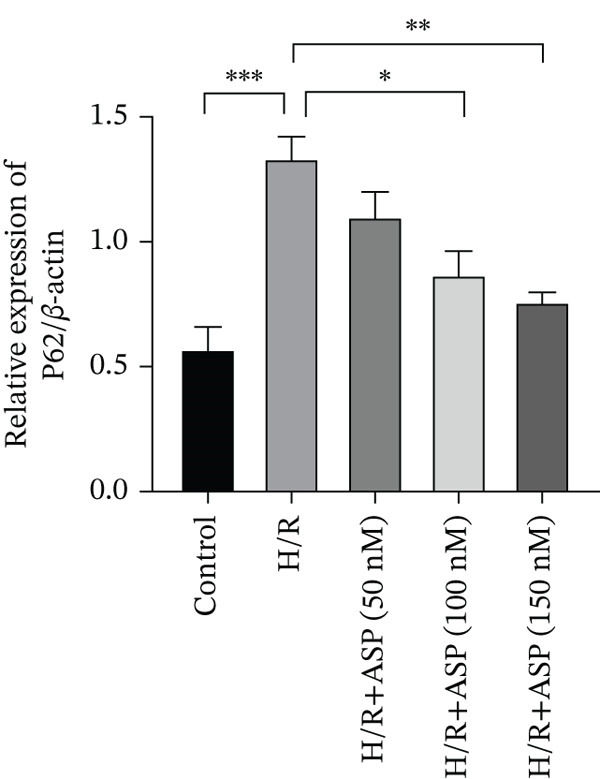
(b)

**Figure:**
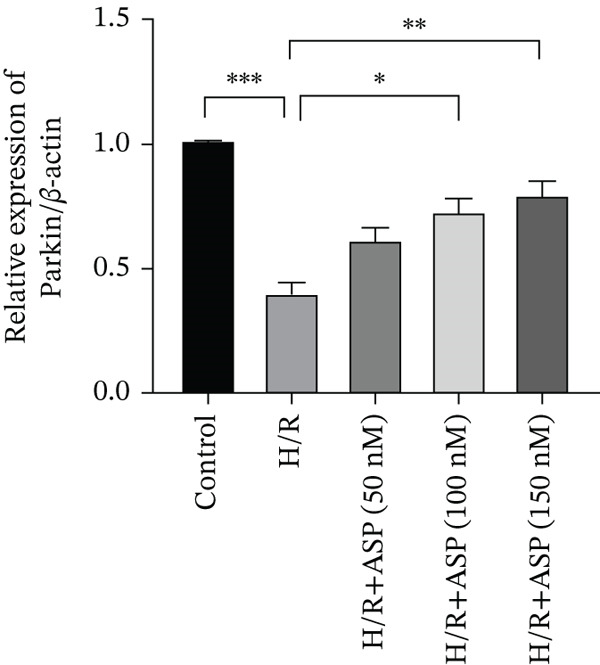
(c)

**Figure:**
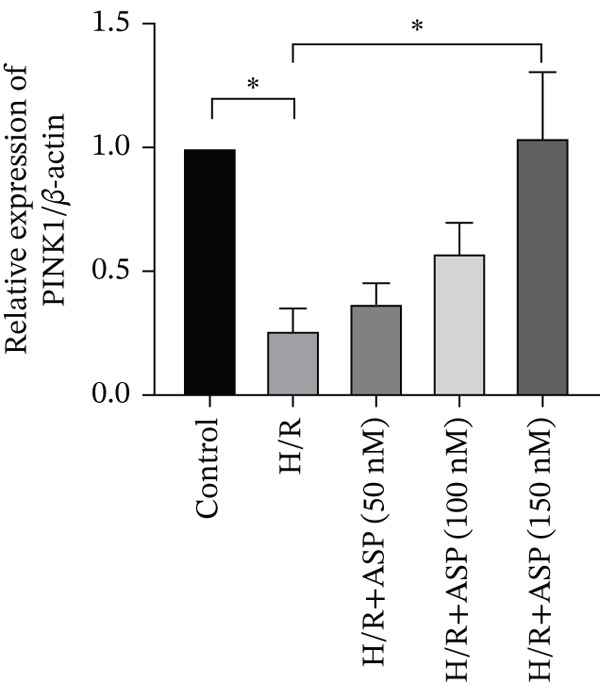
(d)

**Figure:**
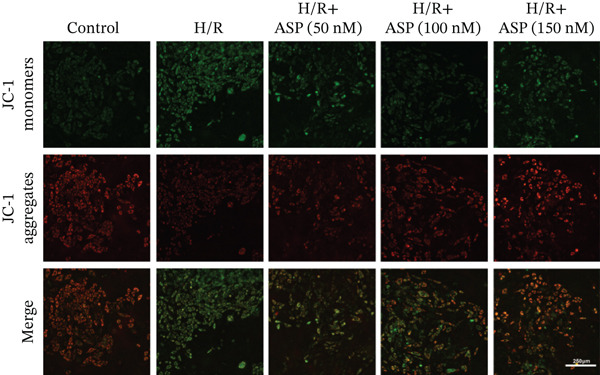
(e)

**Figure:**
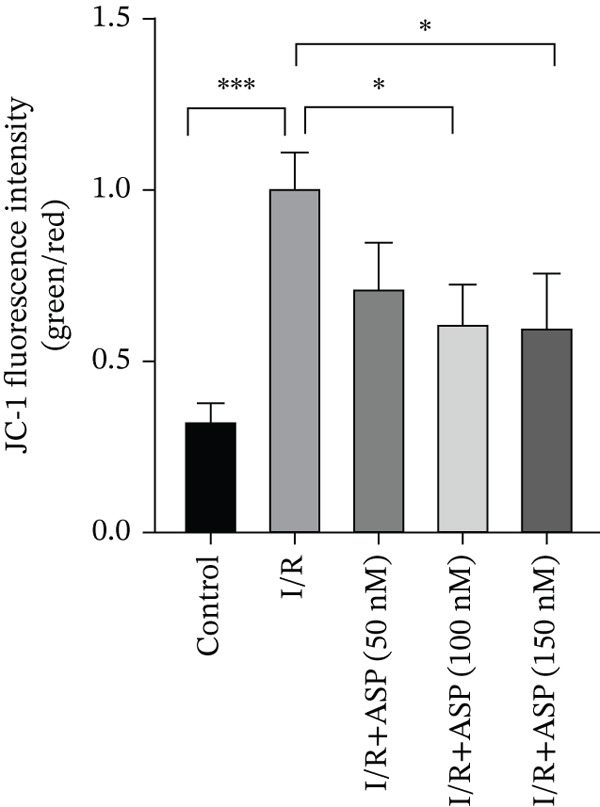
(f)

### 3.6. The Protective Effect of ASP on the Decline in Cell Viability and Ferroptosis Induced by H/R in H9C2 Cells Is Abolished Upon the Inhibition of Mitophagy

We pretreated H9C2 cells with 100 nM ASP and concurrently treated them with the mitophagy inhibitor Mdivi‐1 (50 *μ*M) to suppress mitophagy. To confirm the suppression of mitophagy, we reassessed the levels of PINK1 protein and the mitochondrial membrane potential. We observed that Mdivi‐1 promoted the expression of P62 protein and inhibited the increase in PINK1 and Parkin protein levels (Figures 6a, 6c, and 6d) that were previously promoted by ASP and enhanced green fluorescence (Figure 6e,f), which indicates a decrease in mitochondrial membrane potential. Additionally, Mdivi‐1 abrogated the protective effect of ASP on cell viability (Figure 6g,h) and increased the production of ROS, which had been suppressed by ASP following H/R injury (Figure 6i,k). Furthermore, we discovered that the inhibition of mitophagy also counteracted the protective effect of ASP on ferroptosis in H9C2 cells post‐H/R injury, as evidenced by a decrease in GSH levels (Figure 6l) and the expression of GPX4 and SLC7A11 proteins (Figure 6n, Figure 6o,p), and an increase in cellular Fe^2+^ levels (Figure 6j,m) compared to previous conditions. These findings suggest that ASP protects against the decline in cell viability and ferroptosis induced by H/R injury in H9C2 cells by promoting mitophagy.

Figure 6ASP participates in the alteration of cell viability and ferroptosis induced by H/R in H9C2 cells by affecting mitophagy. (a, b) The expression levels of P62 were measured by western blotting. (a, c) The expression levels of Parkin were measured by western blotting. (a, d) The expression levels of PINK1 were measured by western blotting. (e) Fluorescence of H9C2 cell mitochondria JC‐1 staining (magnification 200x, scale bar 250 *μ*m). (f) Ratio of JC‐1 staining green/red fluorescence. (g) LDH‐mediated cell death assay. (h) CCK‐8 cell viability assay. (i, k) Intracellular ROS concentration (magnification 200x, scale bar 250 *μ*m). (j, m) Intracellular Fe^2+^ content was determined using corresponding quantitative assay kits to observe the effect of ASP at a concentration gradient on Fe^2+^ in H9C2 cells following H/R injury (magnification 200x, scale bar 250 *μ*m). (l) Relative expression of cellular GSH. (n, o) The expression levels of SLC7A11 were measured by western blotting. (n, p) The expression levels of GPX4 were measured by western blotting. Data (*n* = 3) are expressed as mean ± SD, with significance indicated at *p* < 0.05,  ^∗^ *p* < 0.05,  ^∗∗^ *p* < 0.01, and  ^∗∗∗^ *p* < 0.001.

**Figure:**
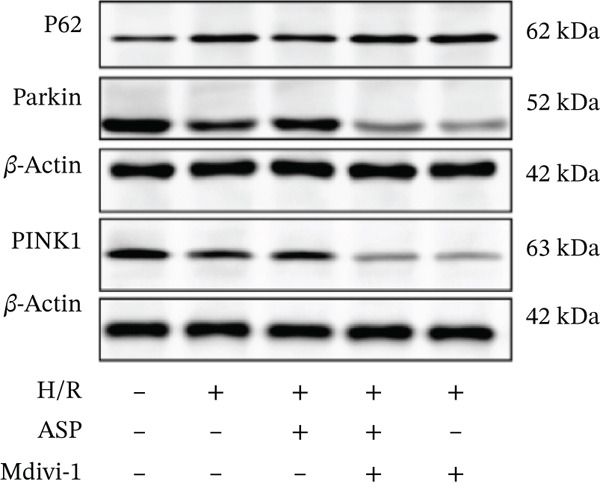
(a)

**Figure:**
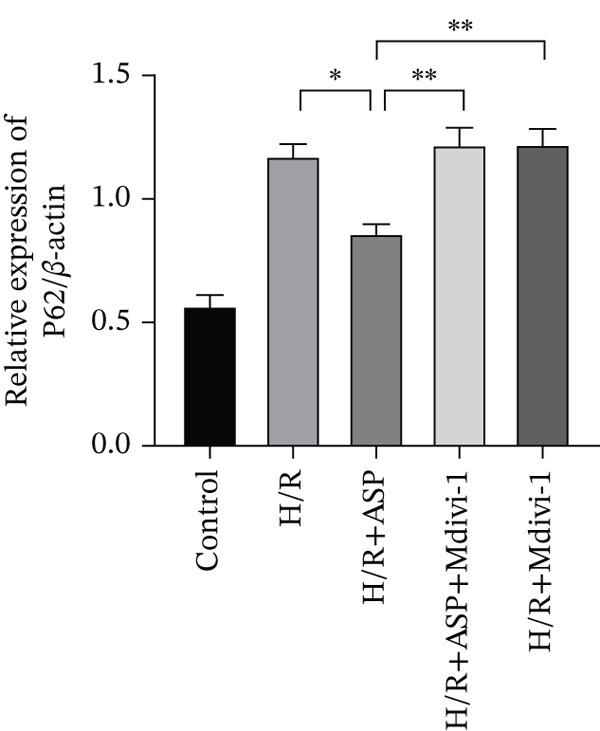
(b)

**Figure:**
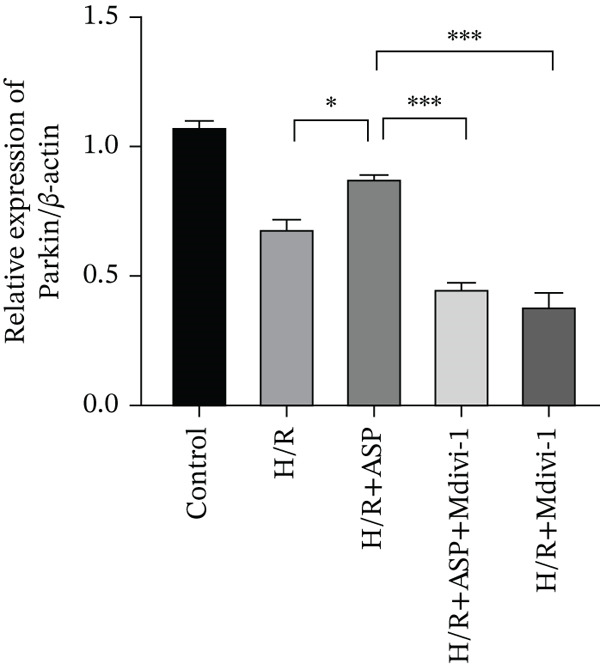
(c)

**Figure:**
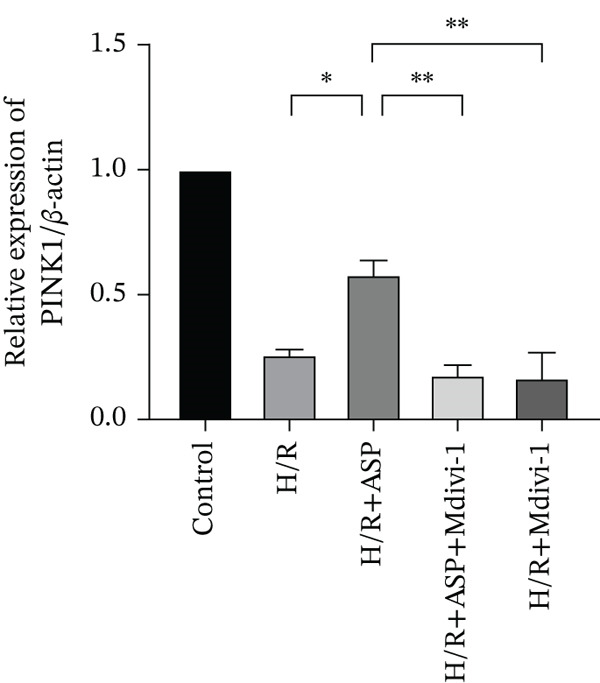
(d)

**Figure:**
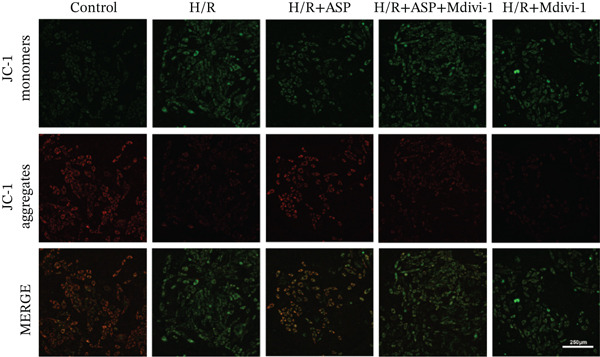
(e)

**Figure:**
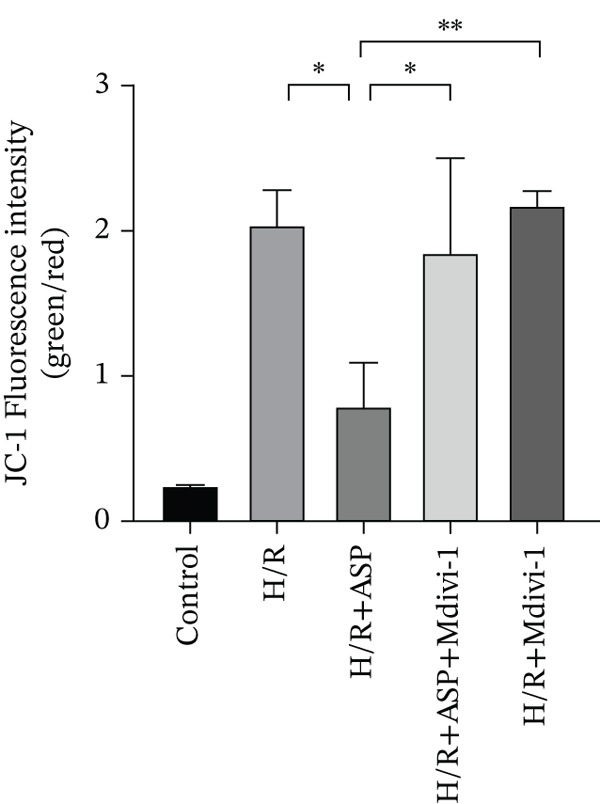
(f)

**Figure:**
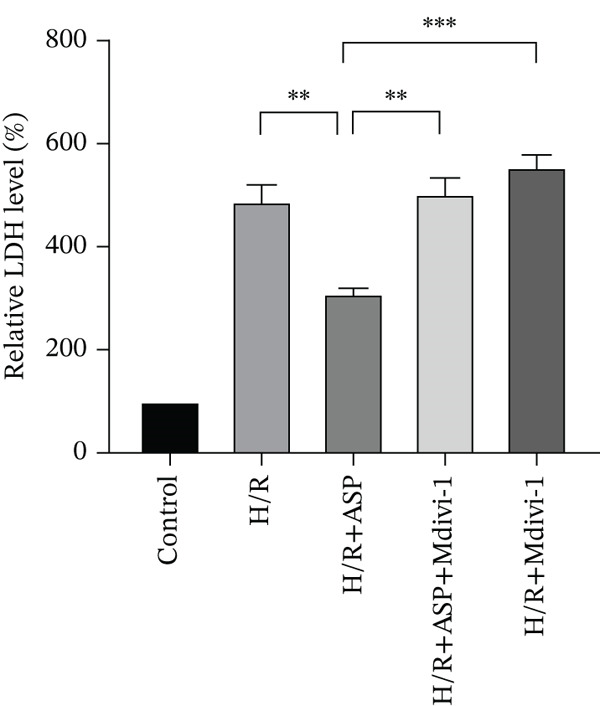
(g)

**Figure:**
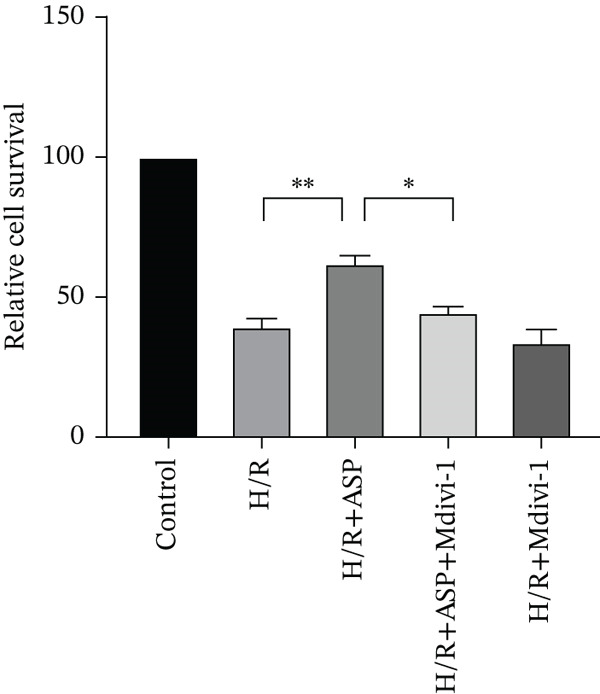
(h)

**Figure:**
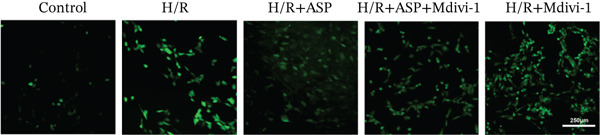
(i)

**Figure:**
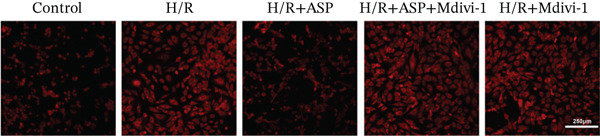
(j)

**Figure:**
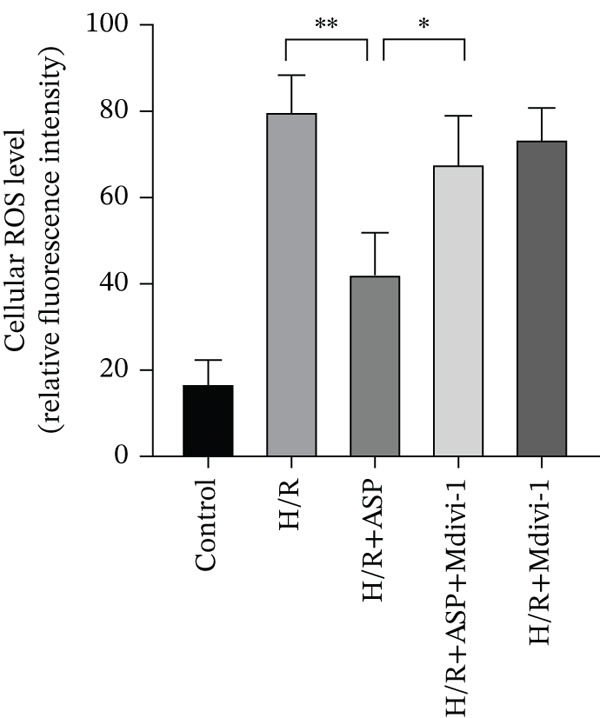
(k)

**Figure:**
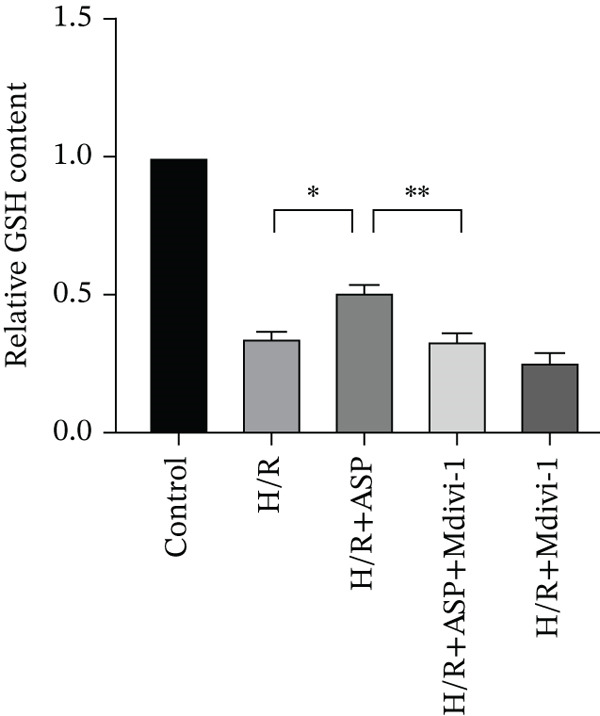
(l)

**Figure:**
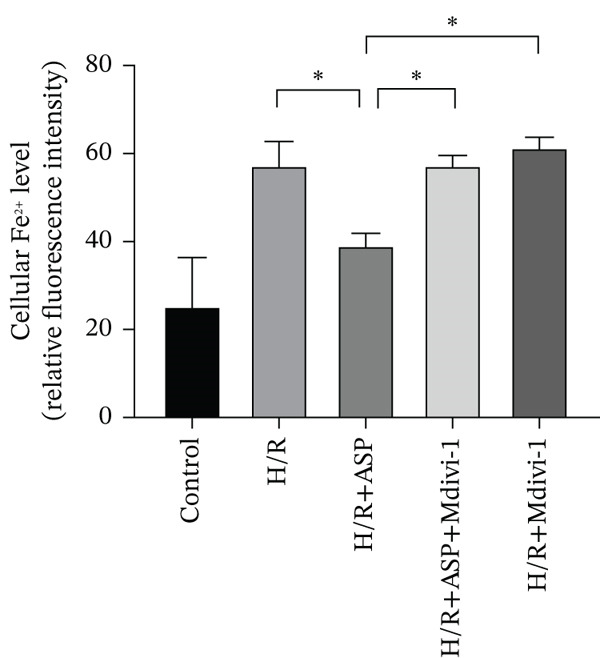
(m)

**Figure:**
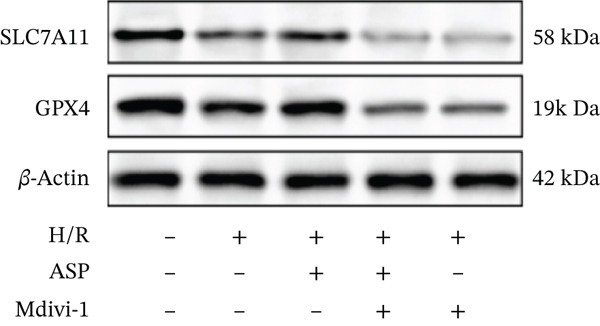
(n)

**Figure:**
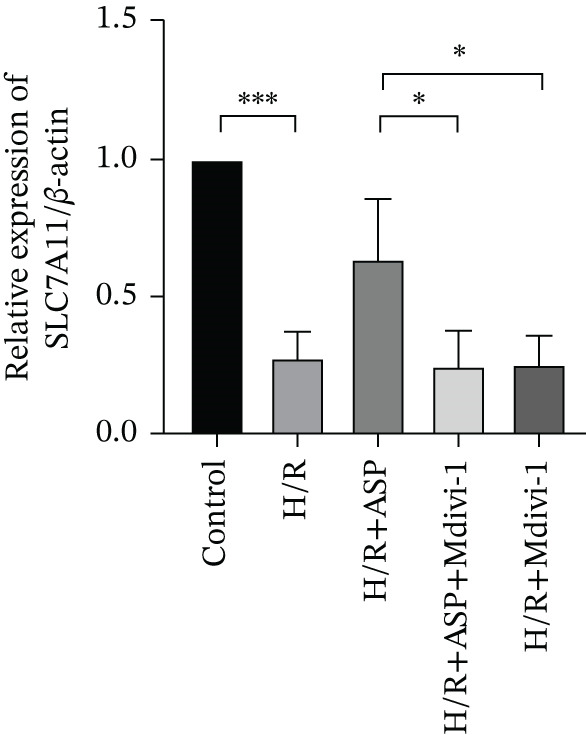
(o)

**Figure:**
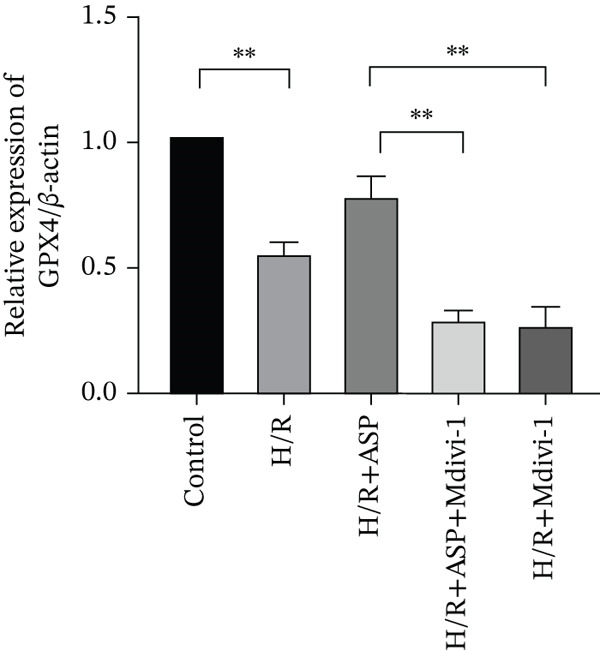
(p)

## 4. Discussion

In this study, we applied ASP and the mitophagy inhibitor Mdivi‐1 to the H/R model of H9C2 cells and observed changes in mitophagy and ferroptosis indicators. We provided multiple lines of evidence demonstrating that ASP plays an inhibitory role in H/R‐induced ferroptosis of cardiomyocytes. Furthermore, we found that the addition of Mdivi‐1 to ASP can diminish this inhibitory effect. Mechanistically, ASP affects the PINK1 protein and promotes mitophagy activation in H9C2 cells after H/R, ultimately inhibiting ferroptosis through the SLC7A11‐GPX4 axis. In addition, we discovered that ASP mitigates the accumulation of ROS and Fe^2+^ in H9C2 cells induced by H/R, alleviates the consumption of GSH, which also helps to inhibit the occurrence of ferroptosis in cells. In summary, we believe that ASP can inhibit ferroptosis induced by H/R and plays a protective role, showing potential for practical application in severe cardiovascular diseases such as MIRI.

ASP, a newly discovered hormone, has many cardiac‐related mechanisms that warrant further exploration. An increasing body of evidence in recent years has suggested a protective role for ASP in the heart. Clinical studies have demonstrated that ASP improves the long‐term prognosis of patients with dilated cardiomyopathy and reduces the risk of heart failure events, attributing these effects to enhanced mitochondrial function in cardiomyocytes [9]. Research in muscle tissue has also shown that ASP can increase PGC‐1*α* levels [25], a regulator of mitochondrial biogenesis and the quality control mechanisms of mitochondria, including fission, fusion, and mitophagy [26]. ASP also protects MSCs from hydrogen peroxide (H_2_O_2_)‐induced apoptosis and ROS damage by activating the extracellular signal‐regulated kinase 1/2 pathway and upregulating the antioxidant protein superoxide dismutase 2 [6]. In this study, we observed that pretreatment of H9C2 cells with increasing concentrations of ASP resulted in elevated levels of cellular viability following H/R injury, thereby providing further evidence for the protective role of ASP against H/R‐induced injury in cardiomyocytes.

Ferroptosis, a novel form of regulated cell death, is intimately associated with oxidative stress and is characterized by the production of ROS and lipid peroxidation [27]. It can be triggered by the loss or deficiency of activity of the selenoenzyme GPX4 [28]. Emerging evidence suggests that ferroptosis is implicated in several pathological processes, including organ I/R injury, myocardial infarction, neurodegeneration, and carcinogenesis [17, 29]. GPX4 has been identified as a significant therapeutic target for MI/R, a notion that has been corroborated by numerous studies [29]. Intracellular iron overload can trigger an excess production of ROS through the Fenton reaction, initiating lipid peroxidation reactions with polyunsaturated fatty acids within biomembranes and ultimately resulting in ferroptosis in cardiomyocytes [30]. Previously, no study has established a relationship between ASP and ferroptosis. In this experiment, we induced ferroptosis in cells using a MI/R model. We observed that increasing concentrations of ASP treatment elevated the protein expression levels of GPX4 and SLC7A11 in H9C2 cells subjected to H/R, while concurrently reducing the intracellular iron content and ROS levels. This indicates that ASP enhances the antioxidant capacity of H9C2 cells and exerts an inhibitory effect on cellular ferroptosis.

Mitophagy, a critical pathway in selective autophagy [31], plays a vital role in the cellular degradation of damaged mitochondria. In recent years, an increasing body of evidence has suggested that modulating mitophagy plays a significant role in alleviating MIRI [32, 33]. Additionally, other studies have indicated that ASP can induce mitophagy in adipocytes [10]. Hypoxic damage leads to impaired mitochondrial respiration in cells; however, treatment with ASP results in a significant recovery of mitochondrial function [9]. One of the primary sources of intracellular ROS is the mitochondria [34]. Mitophagy limits mtROS production by removing the aged and damaged mitochondria via the specific sequestration and engulfment of mitochondria in lysosome [35]. This study demonstrates that ASP inhibits the decline of mitochondrial membrane potential and promotes mitophagy in cells via PINK1, thereby reversing the damage caused by I/R injury. The protective effect of ASP on mitochondria also reverses the oxidative stress damage induced by the burst of ROS during reperfusion and interrupts the transmission of death signals, thereby maintaining the energy supply of cardiomyocytes. The pathway via which ASP promotes mitophagy requires further exploration. Available evidence indicates that suppressing ASP activates p38, whereas inhibition of p38 promotes PINK1/Parkin‐mediated mitophagy [11, 36]. Given that p38 is known to interact with both ASP and mitophagy, it may serve as a mediator linking ASP to mitophagy. Moderate mitophagy can remove damaged mitochondria without significantly degrading healthy mitochondria, helping to maintain energy metabolism and cellular function, which is crucial for maintaining cellular homeostasis [37] If mitophagy is excessive, it may lead to the degradation of a large number of healthy mitochondria, resulting in insufficient energy supply, cellular dysfunction, and even cell death [38]. By measuring cell viability, ROS levels, and mitochondrial membrane potential, we found that after applying ASP to promote mitophagy, cell viability increased, ROS levels decreased, and mitochondrial function improved. This suggests that the mitophagy induced by ASP is at an optimal, protective level. In contrast, excessive mitophagy typically leads to the opposite results, including a decrease in mitochondrial membrane potential, increased ROS levels, and reduced cell viability.

In typical circumstances, mitophagy aids in sustaining mitochondrial homeostasis by eliminating impaired mitochondria, consequently decreasing the generation of ROS and forestalling lipid peroxidation. Nevertheless, when mitochondrial functionality is compromised, as seen during H/R injury, there is an augmentation in ROS production, which triggers lipid peroxidation and ferroptosis. For instance, Lin et al. found that BNIP3‐mediated and PINK1‐PARK2‐mediated mitophagy protects renal tubular epithelial cells from cisplatin‐induced ferroptosis via the ROS/HO‐1/GPX4 axis [39]. Similarly, Chen et al. showed that the promotion of Parkin‐dependent mitophagy inhibits ferroptosis in cardiomyocytes under diabetic cardiomyopathy conditions [40]. Wang et al. demonstrated that mitophagy can promote GSH, a crucial antioxidant that neutralizes ROS and prevents lipid peroxidation [41]. Our results suggest that ASP enhances PINK1/Parkin‐mediated mitophagy, restores damaged mitochondrial function, reduces the elevated ROS and ferrous ion levels induced by H/R, and ultimately inhibits ferroptosis, thereby restoring cell viability. After inhibition of mitophagy with Mdivi‐1, the protective effect of ASP was significantly weakened: intracellular ROS levels increased, GPX4 and SLC7A11 expression decreased, intracellular ferrous ions increased, GSH levels decreased, and the ferroptosis inhibition effect disappeared. This supports the notion that mitophagy inhibits ferroptosis by maintaining iron homeostasis and reducing ROS.

## 5. Conclusions

We have validated the protective effect of ASP on cardiomyocytes subsequent to H/R injury and have discovered that ASP can inhibit ferroptosis and promote mitophagy in cardiomyocytes following H/R injury. The underlying mechanism of this effect may involve the promotion of PINK1‐associated mitophagy in cardiomyocytes post‐H/R injury by ASP, thereby suppressing ferroptosis. Further evidence is required to elucidate the specific mechanisms underlying the relationship between ASP and ferroptosis.

**Nomenclature**
FBN1: Fibrillin 1
ASP: asprosin
WGCNA: gene coexpression network analysis
ROS: reactive oxygen species
CCK‐8: Cell Counting Kit‐8
LDH: lactate dehydrogenase
PINK1: phosphatase and tensin homolog–induced kinase 1
GPX4: glutathione peroxidase 4
SLC7A11: solute carrier family 7 member 11
Fer‐1: Ferrostatin‐1
Mdivi‐1: mitochondrial division Inhibitor 1
GSH: Glutathione
MIRI: myocardial ischemia–reperfusion injury
I/R: ischemia/reperfusion
H/R: hypoxia/reoxygenation
AMI: acute myocardial infarction
MSCs: mesenchymal stem cells
DCM: dilated cardiomyopathy
GEO: Gene Expression Omnibus
LAD: left anterior descending artery
FerrDb: ferroptosis database
BP: biological process
MF: molecular function
CC: cellular component
OD: optical density
SEM: standard error of the mean
MAD: median absolute deviation
**MAPK**
: mitogen‐activated protein kinase

## Author Contributions

X.W. and X.Z. wrote the draft; H.T., S.D., and X.S. performed the data analysis and data presentation; X.L. and B.L. reviewed, edited, and proofed the manuscript. All authors contributed to the article. X.W. and X.Z. contributed equally to this work.

## Funding

This work was supported by the Natural Science Foundation of Shandong Province, 10.13039/501100007129, No. ZR2023MH268.

## Disclosure

All authors approved the submitted version.

## Ethics Statement

The authors have nothing to report.

## Consent

The authors have nothing to report.

## Conflicts of Interest

The authors declare no conflicts of interest.

## Data Availability

The datasets used and analyzed during the current study are available from the corresponding author on reasonable request.
