# Correction to “Asprosin Protects H9C2 Cells From Ferroptosis Following Hypoxia/Reoxygenation by Promoting Mitophagy”

**DOI:** 10.1155/cdr/9810149

**Published:** 2026-07-13

X. Wang, X. Zhang, H. Tang, et al., “Asprosin Protects H9C2 Cells From Ferroptosis Following Hypoxia/Reoxygenation by Promoting Mitophagy,” *Cardiovascular Therapeutics* 2026 (2026): 8401037, https://doi.org/10.1155/cdr/8401037

In this above article, there was an error in the affiliation. The correct affiliation list and author affiliations are shown below.

Xiangkun Wang^1^, Xiaoyu Zhang^2^, Huaiguang Tang^1^, Xiaojuan Su^3^, Shutong Ding^3^, Xuelian Li^4^, and Bingong Li^5^

^1^Department of Cardiology, Qingdao Municipal Hospital, Qingdao University, Qingdao, China

^2^Department of Neurology, The Second Affiliated Hospital, Harbin Medical University, Harbin, Heilongjiang Province, China

^3^School of Clinical Medicine, Shandong Second Medical University, Weifang, Shandong, China

^4^Department of Cardiology, Union Hospital, Tongji Medical College, Huazhong University of Science and Technology, Wuhan, China

^5^Department of Cardiology, Qingdao Hospital of Rehabilitation University, East Hospital of Qingdao Municipal Hospital, Qingdao, Shandong, China

We apologize for this error.

